# Microbial profiling of five traditional Chinese medicines: culture-based identification vs. 16S rRNA sequencing

**DOI:** 10.1099/jmm.0.002137

**Published:** 2026-03-31

**Authors:** Banzhan Ruan, Shi Li, Caixia Chen, Bingshu Wang, Xueyin Wang, Le Du, Qibing Liang, Chunyan Feng, Shaojiang Zheng

**Affiliations:** Key Laboratory of Emergency and Trauma of Ministry of Education, Engineering Research Center for Hainan Biological Sample Resources of Major Diseases, Hainan Branch of National Clinical Research Center for Cancer1, The First Affiliated Hospital, Hainan Medical University, Haikou 570102, PR China; Department of Biology, School of Basic Medicine2, Hainan Medical University, Haikou 571199, PR China; Department of Urology, Key Laboratory of Clinical Laboratory Diagnosis and Translational Research of Zhejiang Province, The First Affiliated Hospital of Wenzhou Medical University, Wenzhou Zhejiang 3250053, PR China; Hainan Inspection and Testing Institute4, Haikou 570311, PR China; Department of Pathology5, The Second Affiliated Hospital of Hainan Medical University, Haikou 570311, PR China; 6 Hainan Center for Drug Inspection, Haikou 571199, PR China

**Keywords:** traditional Chinese medicines, microbial contamination, microbial profiling, 16S rRNA sequencing, VITEK, *Enterobacter*

## Abstract

**Introduction.** Traditional Chinese medicines (TCMs) may carry microbial hazards, yet conventional inspection methods often underestimate their diversity and risks.

**Hypothesis.** Culture-based methods may overlook significant microbial diversity and potential pathogens compared with sequencing approaches.

**Aim.** This study aimed to compare culture-based identification (VITEK-2) with 16S rRNA high-throughput sequencing for microbial detection in selected TCMs.

**Methodology.** Thirty batches of five southern TCM samples, including *Areca catechu*, *Alpinia oxyphylla*, *Eugenia caryophyllata*, *Pogostemon cablin* and *Morinda officinalis*, were analysed for microbial contamination according to the ‘Pharmacopoeia of the People’s Republic of China’ (2020 Edition). Culture-based isolates were identified using VITEK-2, and microbial communities were profiled by 16S rRNA sequencing.

**Results.** Culture-based methods identified bile salt-resistant Gram-negative bacteria in 12 out of 30 batches (40%), comprising seven genera spanning two phyla, including *Enterobacter cloacae*, *Pseudomonas aeruginosa*, *Cronobacter sakazakii* and *Klebsiella pneumoniae*. Sequencing identified 795 genera across 26 phyla, revealing far greater diversity, though DNA signatures of some cultured taxa (e.g. *Enterobacter* in *M. officinalis* and *Leclercia* in *Areca catechu*) were undetected. Microbial composition varied significantly among TCMs.

**Conclusion.** Sequencing uncovered substantially broader microbial diversity than culture-based methods, while both approaches identified pathogenic and opportunistic microbes. These findings emphasize the critical need for more rigorous microbial surveillance and stringent quality control of TCMs to protect public health.

## Data Summary

The 16S rRNA sequencing data have been deposited in the National Center for Biotechnology Information (NCBI) Sequence Read Archive under BioProject PRJNA1133095. The individual SRA accession numbers (30 runs) are provided in Table S1 (available in the online Supplementary Material).

## Introduction

Traditional Chinese medicine (TCM) is a valuable heritage of the Chinese nation, with medicinal herbs serving as the material foundation of this traditional medical system. Traditional medicines from South China, known as southern TCMs, hold a significant position within China’s medicinal herb industry [1]. These medicinal herbs primarily grow in the subtropical and tropical regions south of the Yangtze River, where the warm, humid climate and fertile soil provide ideal natural conditions for their growth [2]. In this study, five representative herbs *– Areca catechu*, *Alpinia oxyphylla*, *Eugenia caryophyllata* (also known as Clove), *Pogostemon cablin* (also known as Patchouli) and *Morinda officinalis* – were included for investigation due to their high incidence of contamination, extensive usage and substantial economic value. Among these, *Areca catechu*, *M. officinalis* and *Alpinia oxyphylla* are among the four major southern medicinal herbs and are widely used in traditional Chinese medicine [3]. *P. cablin* and *E. caryophyllata*, in addition to being important spices extensively used in the food and cosmetic industries, possess unique pharmacological properties, significant clinical application value and high market circulation [4,7]. Meanwhile, multiple reports have indicated that *Areca catechu* and *Alpinia oxyphylla* are associated with microbial contamination issues, particularly involving fungi and toxins [8,9]; *M. officinalis* and *P. cablin* present potential risks [10,11].

Microbial contamination of traditional Chinese medicines is a common problem that cannot be ignored. Microbial contamination in traditional Chinese medicinal materials includes bacteria, fungi, viruses and other micro-organisms. Such contamination stands as a significant contributor to medication-associated infections and inconsistent therapeutic outcomes [12]. Given that these materials undergo processes like decoction and boiling before administration, certain heat-resistant micro-organisms or bacterial endotoxins may persist, posing risks of gastrointestinal and respiratory infections upon ingestion, or enter the blood circulation system upon intravenous infusion [13]. Furthermore, microbial proliferation within these materials can lead to the formation of metabolites. Some of these metabolites may be toxic or allergenic [14]. These contaminants jeopardize the medicinal quality, potentially causing products to fall short of microbial standards outlined in pharmacopoeias, thereby compromising their approval rates and reliability.

However, currently used microbial control methods have many limitations. Various national pharmacopoeias outline microbiological testing methods and limits for natural medicinal raw materials. In the 2020 edition of the 'Chinese Pharmacopoeia’, the conventional plate method remains the standard for assessing microbial limits in traditional Chinese medicinal pieces [15]. Although recent studies indicate that over one-third of microbial taxa may be culturable [16], culture-based methods still capture only a fraction of total microbial diversity, underscoring their inherent limitations.

Given these limitations, high-throughput sequencing technology has emerged as a pivotal tool for comprehensive microbial analysis due to its efficiency, maturity and cost-effectiveness [17,20]. The 16S rRNA sequence comprises both conserved regions and highly variable regions. The conserved regions exhibit minimal differences among microbial species, while the highly variable regions are genus- or species-specific and show distinct variations depending on phylogenetic relationships. Sequencing the 16S rRNA allows for the clarification of the taxonomic relationships of microbial populations from an evolutionary genetics perspective [21], which makes it possible to comprehensively analyse microbial communities that contaminate traditional Chinese medicines and decoctions [22].

By directly comparing culture-based identification (VITEK-2) and high-throughput 16S rRNA sequencing, this study elucidated both the composition and extent of microbial contamination in traditional Chinese medicinal materials. The comparison highlights the strengths and limitations of each approach and provides critical data to support early warning and risk management strategies, ultimately helping to prevent contamination and safeguard clinical safety and public health.

## Methods

### Traditional Chinese medicines

This study investigated five typical southern medicinal materials: *Areca catechu*, *Alpinia oxyphylla*, *E. caryophyllata* (also known as Clove), *P. cablin* (also known as Patchouli) and *M. officinalis* (*M. officinalis*). A total of 30 batches, comprising 6 batches of each medicinal material, were collected. Each ‘batch’ represents an independent lot from a distinct producer and/or harvest, obtained from various wholesale medicinal material markets across multiple provinces *– Areca catechu* from Hainan and Yunnan, *Alpinia oxyphylla* from Hainan and Guangdong, *E. caryophyllata* from Hainan and Guangdong, * P. cablin* from Hainan and Guangdong and *M. officinalis* from Hainan and Guangxi – ensuring that no batch originated from a single production site. All batches were analysed individually without pooling to preserve batch-specific variation. In detail, a minimum of 50 g per batch was sampled, ground individually and thoroughly mixed. From the blended sample, 25 g of powder was weighed and combined with 225 ml of 0.9% sterile sodium chloride solution. After shaking well, the mixture was allowed to stand for 5 min, and the resulting supernatant was tested at a 1:5 or 1:10 dilution.

### Culture-based Identification

For all five selected traditional Chinese medicinal materials, the culture-based identification was conducted in accordance with the ‘Controlled Bacteria Inspection Method’ from the 2020 Edition of the Chinese Pharmacopoeia, specifically method 1108, focusing on detecting bile salt-resistant Gram-negative bacteria and *Salmonella*. Briefly, the examination of bile-resistant Gram-negative bacteria was performed as follows. Weigh 25 g of the test sample, and add it to 225 ml of tryptic soy broth (TSB) liquid medium (Huankai, Guangdong, China), mixing well to create a 1:10 test solution. Pre-culture the solution at 25 °C for 2 h. Then, inoculate 10 ml of the pre-culture into 100 ml of enteric bacteria enrichment broth (Huankai, Guangdong, China) and incubate at 32.5 °C for 48 h. Finally, streak the inoculum onto a purple–red bile glucose agar plate (Huankai, Guangdong, China) and incubate at 32.5 °C for 24 h. Different from the examination of bile-resistant Gram-negative bacteria, *Salmonella* inspection was done as follows. Weigh 10 g of the sample, and inoculate it into 100 ml of TSB liquid medium (Huankai, Guangdong, China). The mixture was incubated at 32.5 °C for 24 h. Transfer 0.1 ml of the culture to 10 ml of RV *Salmonella* enrichment broth (Huankai, Guangdong, China), and incubate again at 32.5 °C for 24 h. Finally, streak a small amount of the RV *Salmonella* enrichment culture onto a xylose lysine deoxycholate agar plate (Huankai, Guangdong, China), and incubate at 32.5 °C for 48 h. Microbial colonies with varying morphological characteristics that grew on purple bile glucose agar and xylose lysine deoxycholate agar were selected. These colonies were then streaked onto tryptic soy agar (Huankai, Guangdong, China) for isolation and purification. Each sample was enriched and plated in duplicate (two technical replicates). Positive controls (*Escherichia coli*) and negative controls (uninoculated enrichment broth) were processed in parallel. All culture work (especially operations involving *Salmonella* and *Pseudomonas aeruginosa*) was conducted in a certified BSL-2 laboratory following biosafety guidelines.

Bacterial identification was performed using the VITEK-2 Compact system (bioMérieux, France, version 07.01) with GN identification cards, in strict accordance with the manufacturer’s instructions. The system performed taxonomic assignment by comparing the biochemical biopatterns of the isolate against the proprietary VITEK-2 reference database updated within the software. Results were expressed as a numerical probability (e.g. 99%) and a qualitative confidence level (e.g. ‘Excellent Identification’). Quality control was performed using standard reference strains as per the manufacturer’s recommendations.

### Extraction of genomic DNA, PCR amplification and 16S rRNA sequencing

The DNA extraction, PCR amplification and 16S rRNA gene sequencing were conducted by Majorbio Bio-pharm Technology Co., Ltd. (Shanghai, China). To ensure data integrity, extraction and PCR negative controls were processed in parallel with the samples. These controls exhibited no significant amplification and yielded negligible sequence reads. In detail, the genomic DNA of all five traditional Chinese medicinal samples was extracted and qualified with 1% agarose gel electrophoresis. A nested PCR approach (initial amplification with 799 F-1392R, followed by 799 F-1193R) targeting the bacterial V5–V7 region of the 16S rRNA gene was employed to enhance specificity and minimize host DNA interference as described [23,24]. Briefly, the first round, using the 799 F-1392R primer pair, consisted of 27 cycles, followed by a second round of 13 cycles with the 799 F-1193R primer pair. This process successfully amplified a 394-bp fragment of the V5–V7 region of the 16S rRNA gene from endophytic bacteria. The primer sequences used were 799F (5′-AACMGGATTAGATACCCKG-3′), 1392R (5′-ACGGGCGGTGTGTRC-3′) and 1193R (5′-ACGTCATCCCCACCTTCC-3′). The PCR product was confirmed using 2% agarose gel electrophoresis. Based on preliminary quantitative electrophoresis results, the PCR product was detected and quantified using the QuantiFluor^™^ -ST blue fluorescence quantitative system (Promega). Adapter sequences were incorporated at both ends of the fragment through a second round of PCR with adapter-ligated primers. Subsequently, paired-end sequencing was performed according to the Illumina MiSeq instrument’s instruction manual.

### Statistical and bioinformatics analysis

After the reads obtained by Illumina sequencing were sorted into samples, the paired-end reads were quality-filtered based on sequencing metrics. They were then spliced according to their overlap, resulting in optimized data. This optimized data was further processed using the DADA2 pipeline to perform denoising and generate amplicon sequence variants (ASVs) and their abundance information. Taxonomic assignment of the ASVs was performed using a pre-trained Naive Bayes classifier trained on the silva database (release 138), with a confidence threshold of 0.7. Subsequently, downstream statistical and visualization analyses, including community diversity, composition and species difference analyses, were performed using Majorbio (https://www.majorbio.com/). Additionally, GraphPad Prism 8.0 for Windows (GraphPad software) was used to create alpha diversity index bar graphs. Pairwise comparisons between groups were conducted using the Mann–Whitney *U* test, and *P*-values were adjusted for multiple testing using the Benjamini–Hochberg false discovery rate (FDR) method.

## Results

### Bacteria test

To measure microbial contaminants of five TCMs, streaking from them on Violet Red Bile Glucose agar was performed (Fig. S1). The results of examining 30 batches of Chinese herbal medicine samples for bile-resistant Gram-negative bacteria and *Salmonella* are summarized in Table 1. Bile-resistant Gram-negative bacteria were detected in 12 batches, whereas *Salmonella* was not found in any of the samples.

**Table 1.** Bacteria inspection summary for five traditional Chinese medicines

Sample	Batch no.	Bile-resistant Gram-negative bacteria	*Salmonella*
*Areca catechu*	6	4 (67%)	Not detected
*Alpinia oxyphylla*	6	3 (50%)	Not detected
*E. caryophyllata*	6	1 (17%)	Not detected
*P. cablin*	6	2 (33%)	Not detected
*M. officinalis*	6	2 (33%)	Not detected
**Total**	**30**	**12** (**40%**)	**0**

The prevalence of bile-resistant Gram-negative bacteria varied significantly across different medicinal categories. For instance, *Areca catechu* samples exhibited a 67% detection rate, followed by *Alpinia oxyphylla* samples at 50%. In contrast, *P. cablin* and * M. officinalis* samples both demonstrated equally low rates of 33%, while *E. caryophyllata* showed the lowest rate of 17%.

### Colony identification analysis

Table 2 delineates the identification outcomes of microbial colonies cultured on purple–red bile salt glucose agar (Fig. S1) and xylose lysine deoxycholate agar mediums across various Chinese medicinal materials by the VITEK-2 Compact system (Fig. S2). The contaminants across these materials predominantly belonged to the *Proteobacteria* phylum, spanning three families and eight genera, with *Enterobacteriaceae* exhibiting the highest prevalence and present in all TCM groups.

**Table 2.** Micro-organism identification in five types of Chinese medicinal materials

Sample	Family	Genus	Species
*Areca catechu*	*Enterobacteriaceae*	*Enterobacter*	*Enterobacter cloacae*
		*Cronobacter*	*Cronobacter sakazakii*
		*Leclercia*	*Leclercia adecarboxylata*
		*Klebsiella*	*Klebsiella pneumoniae*
*Alpinia oxyphylla*	*Enterobacteriaceae*	*Enterobacter*	*Enterobacter cloacae*
		*Klebsiella*	*Klebsiella pneumoniae*
*E. caryophyllata*	*Enterobacteriaceae*	*Enterobacter*	*Enterobacter cloacae*
	*Erwiniaceae*	*Pantoea*	*G*enus-specific bacteria*
*P. cablin*	*Enterobacteriaceae*	*Enterobacter* *Klebsiella*	*Enterobacter cloacae* *Klebsiella aerogenes*
			*Klebsiella pneumoniae*
	*Pseudomonadaceae*	*Pseudomonas*	*Pseudomonas aeruginosa* *Pseudomonas putida*
			*Pseudomonas luteola*
*M. officinalis*	*Enterobacteriaceae*	*Enterobacter*	*Enterobacter cloacae*
	*Erwiniaceae*	*Pantoea*	Genus-specific bacteria
	*Lysobacteraceae*	*Stenotrophomonas*	*Stenotrophomonas maltophilia*

* The specific species of micro-organism in the genus have not yet been identified by the VITEK-2 Compact system.

Distinct microbial profiles were discerned among the samples. Family-level analysis revealed that *Pseudomonadaceae* was evident only in *P. cablin* and *M. officinalis*, and *Xanthomonadaceae* was found only in *M. officinalis*. Genus-level insights highlighted *Enterobacter* as the most recurrent. As many as four genera were detected in *Areca catechu*, *P. cablin* and *M. officinalis,* followed by two genera in *Alpinia oxyphylla* and *E. caryophyllata*. Species-level analysis pinpointed six distinct organisms in *P. cablin*, followed by four in *Areca catechu*, with species like *Cronobacter sakazakii*, *Klebsiella pneumoniae* and *Pseudomonas aeruginosa* being notable opportunistic pathogens.

### Microbial community characteristics via high-throughput sequencing

**Fig. 1.**
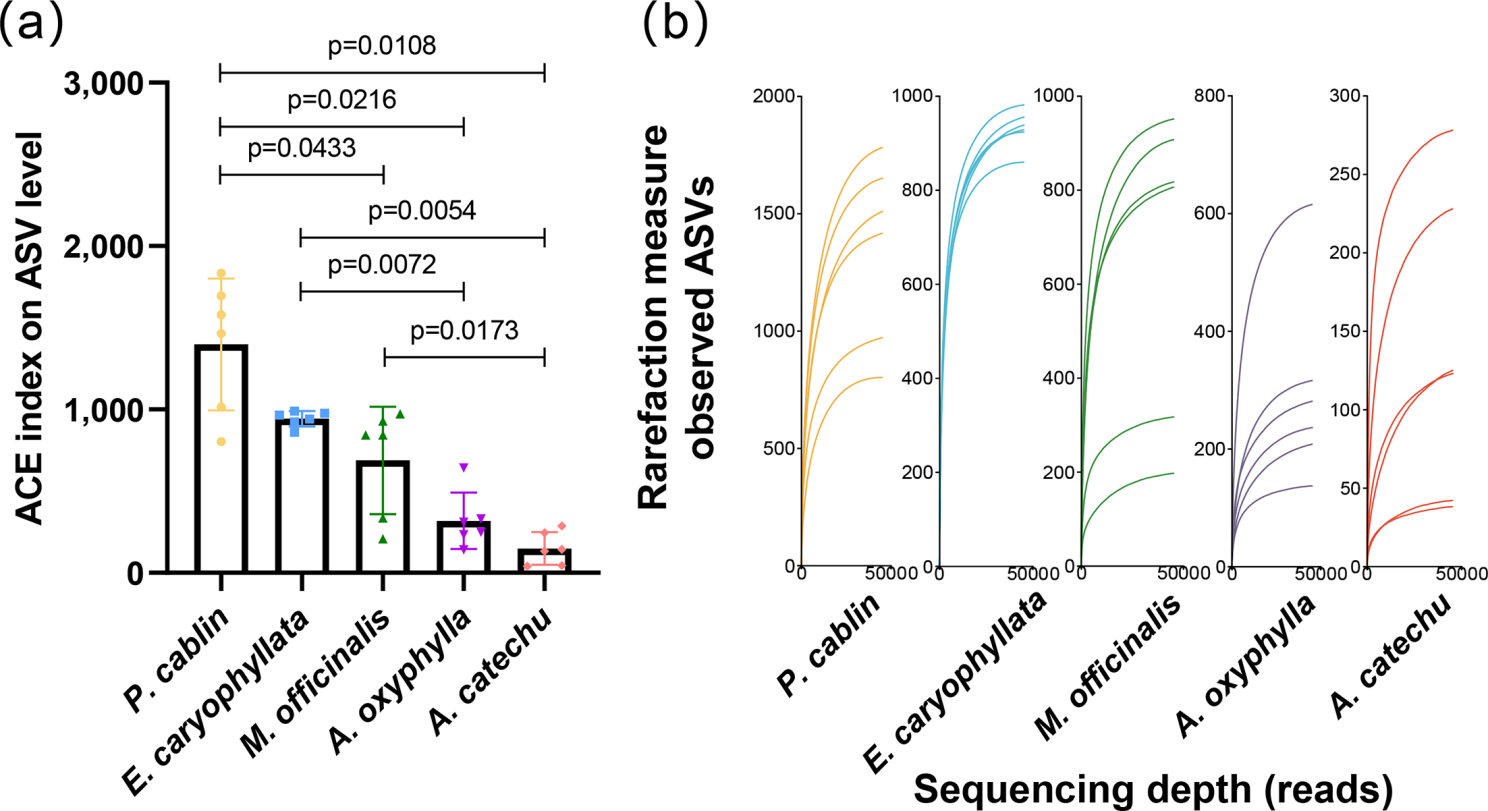
Analysis of microbial colony diversity in five types of Chinese medicinal materials. (**a**) Alpha diversity among five groups based on the ACE index at the ASV level. Data are presented as mean±sem. Pairwise comparisons were conducted using the Mann–Whitney *U* test, with *P-*values adjusted for multiple comparisons using the Benjamini–Hochberg FDR method. Significant differences after FDR correction are indicated (FDR-adjusted, *P<0.05*). (**b**) Rarefaction curve. ASVs are inferred from single DNA sequences obtained from high-throughput analysis of marker genes. This curve assesses whether the sequencing depth is sufficient to capture the full diversity of micro-organisms in TCM samples. It directly reflects the adequacy of the sequencing data volume. When the curve flattens, it indicates that the sequencing depth is appropriate.

In this research, 16S rRNA sequencing was conducted on 30 Chinese herbal medicine samples, yielding 2,263,781 paired-end reads. Utilizing the DADA2 plugin within the QIIME 2 framework, a rigorous de-noising process was executed, encompassing noise filtration, sequence error correction and removal of chimeric reads. Through these processes, high-resolution ASVs were derived for subsequent analyses. Post-DADA2 processing, 1,796,540 sequences were obtained across the samples, averaging between 45,559 and 69,032 sequences per sample and resulting in 20,605 ASVs. Pairwise comparisons of the Abundance-based Coverage Estimator (ACE) index revealed significant differences between groups *P. cabin* and *M. officinalis* (FDR-adjusted, *P*=0.0433), *P. cabin* and *Alpinia oxyphylla* (*P*=0.0216), *P. cabin* and *Areca catechu* (*P*=0.0108), *E. caryophyllata* and *Alpinia oxyphylla* (*P*=0.0072), *E. caryophyllata* and *Areca catechu* (*P*=0.0054) and *M. officinalis* and *Areca catechu* (*P*=0.0173). No significant differences were observed for the remaining comparisons after FDR correction (Fig. 1a).

To ensure the adequacy of the sequencing data for analysis, a preliminary quality control assessment was conducted. All five treatment groups’ rarefaction curves, as seen in Fig. 1b, had a flat state, demonstrating that this study’s sequencing depth is enough to capture the microbial diversity of the samples.

### Analysis of microbial community variations

**Fig. 2.**
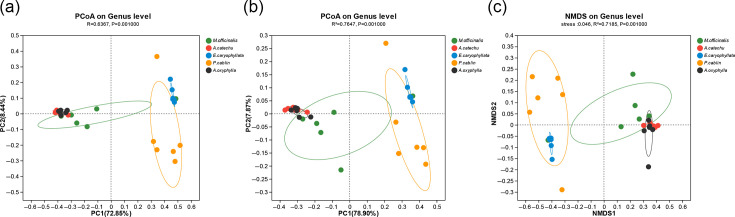
Analysis of microbial community differences. (**a**) PCoA analysis based on Bray–Curtis dissimilarity. *Areca catechu*, *Alpinia oxyphylla* and *M. officinalis* groups were clustered closely along both the PC1 and PC2 axes, while the *E. caryophyllata* and *P. cablin* clusters were separated. (**b**) PCoA analysis using weighted UniFrac distance. PCoA highlighted greater intra-group differences. (**c**) NMDS analysis based on weighted UniFrac distance. A stress value of 0.046 confirmed the applicability of NMDS. The closer association between the *E. caryophyllata* and *P. cablin* groups was observed.

The microbial community compositions across five Chinese medicinal materials were investigated. Using principal coordinate analysis (PCoA) based on Bray–Curtis metrics, distinct clustering was observed. Specifically, the *Areca catechu*, *Alpinia oxyphylla* and *M. officinalis* groups exhibited close proximity along both the PC1 and PC2 axes, forming distinct clusters separate from the *E. caryophyllata* and *P. cablin* groups (Adonis, *R*^2^=0.6367; *P*=0.001). Notably, the *Areca catechu* and *Alpinia oxyphylla* groups displayed significant overlap and were closer than other groups, indicating that their microbial diversity and compositions were closer. In contrast, the *P. cablin* group was farther from the *E. caryophyllata* group, and samples in the *P. cablin* group were more dispersed. An outlier sample within the *P. cablin* group even exhibited closer resemblance to the *E. caryophyllata* group, indicating potential anomalies and reflecting microbial diversity within the *P. cablin* samples (Fig. 2a). These findings were validated by using weighted UniFrac distance in PCoA highlighted even greater intra-group differences, possibly indicative of species evolution patterns (Adonis, *R*^2^=0.7647; *P*=0.001) (Fig. 2b). Additionally, non-metric multidimensional scaling (NMDS) analysis based on weighted UniFrac distance yielded a stress value of 0.046 (below the acceptable threshold of 0.2), confirming the applicability of NMDS and further illustrating the closer association between the *E. caryophyllata* and *P. cablin* groups (NMDS, *R*^2^=0.7185; *P*=0.001) (Fig. 2c).

### Phylum-level micro-organism distribution

**Fig. 3.**
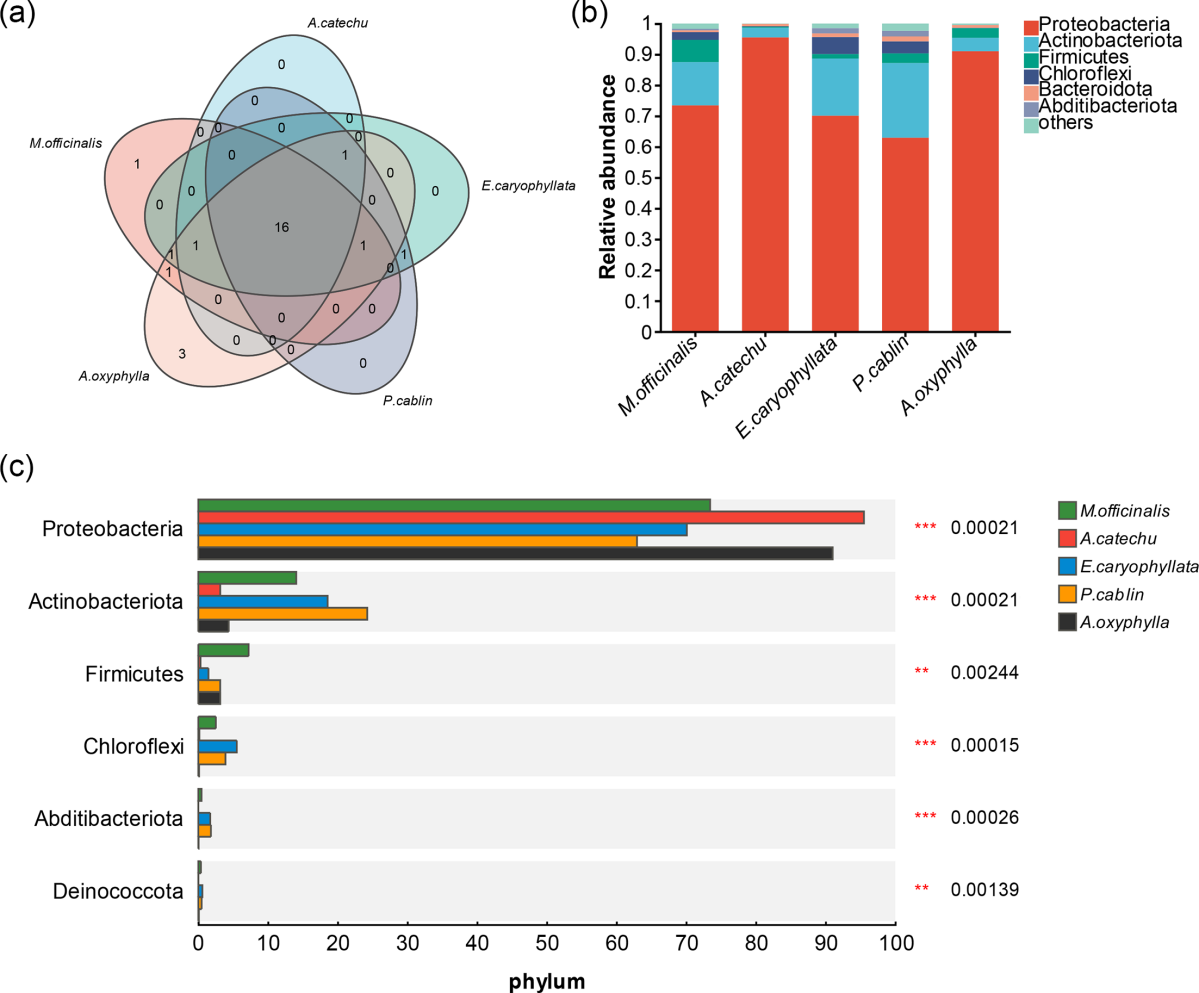
Microbial composition at the phylum level. (**a**) Venn diagram illustrating the clustering of microbial colonies across the five types of Chinese medicinal materials. A total of 16 phyla were found in all groups. Two out of five groups have their own specific phyla, such as one phylum in *M. officinalis* and three phyla in *Alpinia oxyphylla.* (**b**) Bar graph depicting the composition of microbial colonies in each group of Chinese medicinal materials. The top two phyla in each group were *Proteobacteria* and *Actinobacteria.* (**c**) Bar graph showing the distribution of the top six dominant microbial communities across the groups. Certain bacterial phyla exhibited varied distributions among the medicinal materials.

To delineate the bacterial composition within each Chinese medicinal material group, we conducted a statistical assessment of contaminant micro-organism abundance at the phylum level. First, the general and specific micro-organisms enriched in the five groups were analysed. As depicted in Fig. 3a, a total of 16 phyla were found in all groups. Less than half of the groups have their own specific phyla, with one in *M. officinalis* and three in *Alpinia oxyphylla.* Then, we analysed the relative abundance of phyla in each group and found that contaminant bacteria across various samples predominantly belonged to the two phyla *Proteobacteria* and *Actinobacteria* (Fig. 3b). However, certain bacterial groups exhibited varied distributions among the medicinal materials. For instance, *Firmicutes* were predominantly found in *M. officinalis*, followed by *Alpinia oxyphylla*, *P. cablin* and *E. caryophyllata*, while *Chloroflexi* were chiefly present in *M. officinalis*, *E. caryophyllata* and *P. cablin*. A comparative analysis of the top six dominant microbial phyla, i.e. *Proteobacteria*, *Actinobacteria*, *Firmicutes*, *Chloroflexi*, *Abditibacteriota* and *Deinococcota* revealed significant inter-group differences for all six phyla (*P*<0.00244) (Fig. 3c).

### Genus-level distribution of micro-organisms

**Fig. 4.**
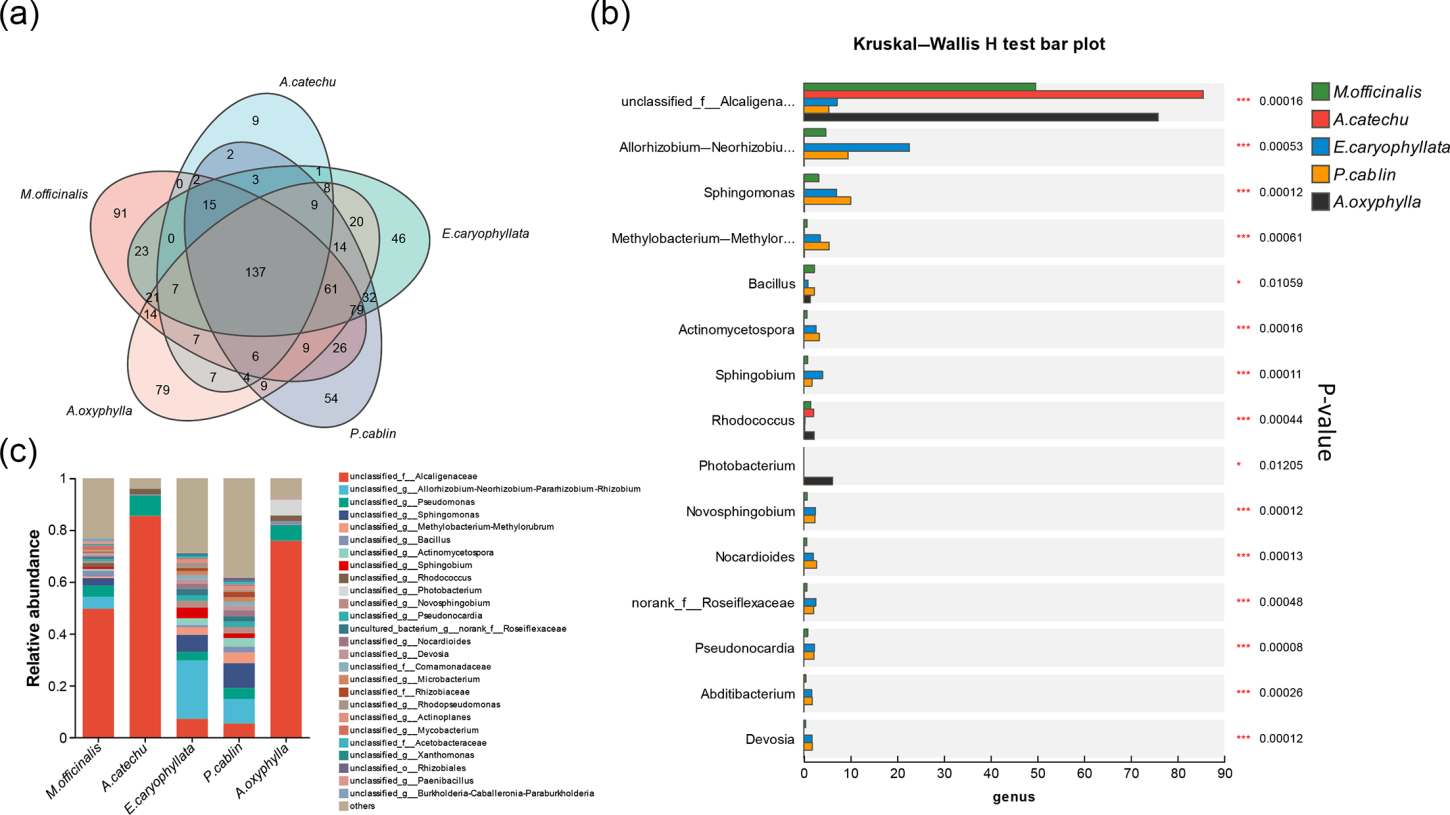
Microbial composition at the genus level. (**a**) Venn diagram illustrating the clustering of microbial colonies across the five types of Chinese medicinal materials. The overlapping area in the middle indicates shared bacteria, and the non-overlapping areas on the sides indicate unique bacteria. (**b**) Bar graph depicting the composition of microbial colonies in each group of Chinese medicinal materials. The relative abundance of micro-organisms of each genus in the five types of Chinese medicinal materials varies greatly. *M. officinalis*, *Areca catechu* and *Alpinia oxyphylla* samples demonstrated a concentrated bacterial composition, while *E. caryophyllata* and *P. cablin* showcased a more diverse bacterial distribution. (**c**) Bar graph showing the distribution of the top 15 dominant microbial communities across the groups. Significant differences in these microbial compositions were observed in each TCM group (‘*’ for *P*<0.05, ‘***’ for *P*<0.001).

A comparative analysis of pollutant microbial genera in different categories of Chinese medicinal materials, as shown in Fig. 4a, revealed that 137 microbial genera are present across all 5 medicinal materials. *M. officinalis* had the highest number of unique bacterial genera with 91, while *Areca catechu* had the fewest with 9. Further analysis indicates significant differences in the dominant genera among the various medicinal materials. Specifically, *M. officinalis*, *Areca catechu* and *Alpinia oxyphylla* samples demonstrated a concentrated bacterial composition, predominantly comprising genera from the *Alcaligenaceae* family, which accounted for over half of the identified species. In contrast, *E. caryophyllata* and *P. cablin* showcased a more diverse bacterial distribution, with the genera *Allorhizobium–Neorhizobium–Pararhizobium–Rhizobium* and *Sphingomonas* emerging as the dominant genera (Fig. 4b). A subsequent analysis revealed significant differences in microbial compositions among TCM groups. The *P*-values for the three dominant genera were all below 0.001. Expanding the analysis to include the top 15 dominant genera, all *P*-values remained below 0.05, with *Pseudonocardia* showing a notably low *P*-value of 0.00008 (Fig. 4c).

### Species variation analysis

**Fig. 5.**
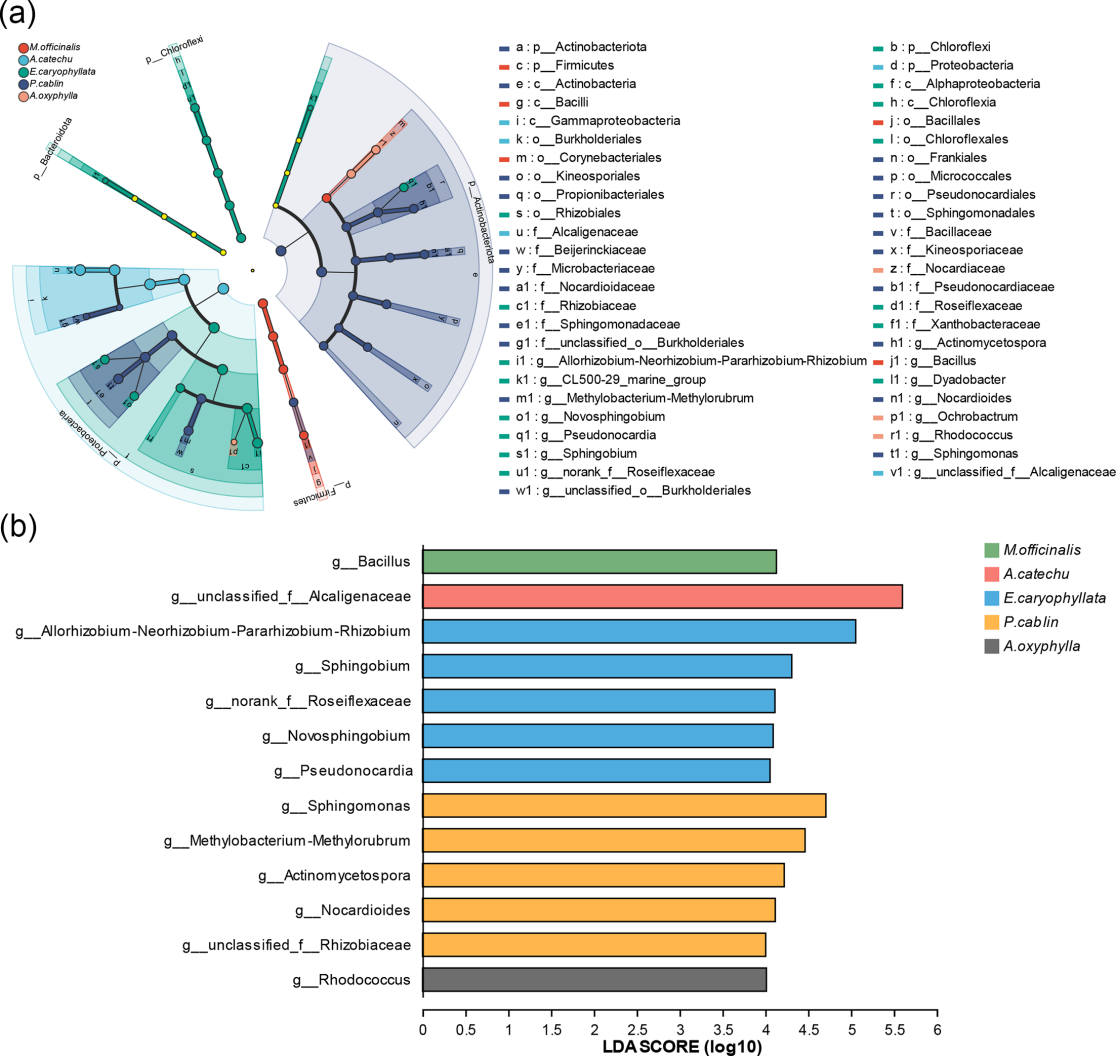
Analysis of species differences. (**a**) Analysis of species differences from the phylum to the genus level. From the outer circle to the centre, each ring represents the kingdom, phylum, class, order, family, genus and species, and each circular node on the ring represents a micro-organism. (**b**) Species with differences at the genus level. There are obvious differences in the specific microbial species among different Chinese medicinal materials groups.

Upon analysing differences across groups from the phylum to genus levels, a total of 56 distinct phyla/genus were identified (Fig. 5a). The *P. cablin* group exhibited the highest diversity, followed by *E. caryophyllata*. In contrast, *Areca catechu*, *M. officinalis* and *Alpinia oxyphylla* displayed more concentrated profiles. Delving deeper into each group’s characteristic microbial genera revealed that *M. officinalis* was predominantly characterized by the phylum *Firmicutes* and the genus *Bacillus. Areca catechu* showcased the family *Alcaligenaceae. E. caryophyllata* featured genera such as *Allorhizobium*, *Sphingobium*, *Novosphingobium* and *Pseudonocardia*, along with the family *Roseiflexaceae. P. cablin* was marked by the detection of DNA signatures from *Sphingomonas* and *Methylobacterium. Alpinia oxyphylla* exhibited *Actinomycetospora*, the genus *Nocardioides* and the family *Rhizobiaceae*, as well as *Rhodococcus* (Fig. 5b). In summary, the specific species composition varied significantly across the different Chinese medicinal material groups.

### Comparison of 16S rRNA sequencing and culture-based identification

Culture-based methods detected seven genera belonging to two phyla, which was markedly fewer than the 795 genera spanning 26 phyla identified by sequencing. DNA signatures of almost all genera isolated by culture (e.g. *Enterobacter*, *Cronobacter*, *Klebsiella*, *Pantoea*, *Pseudomonas* and *Stenotrophomonas*) were also detected by sequencing, except *Enterobacter* in *M. officinalis* and *Leclercia* in *Areca catechu*, which were not recovered. Genera undetected by culture generally showed very low relative abundances in sequencing results (e.g. *Cronobacter* in *E. caryophyllata* accounted for only one read), highlighting the overall consistency between the two methods. Moreover, for certain genera (e.g. *Cronobacter*, *Pantoea*, *Pseudomonas* and *Stenotrophomonas*), sequencing identified their DNA in a greater number of TCMs compared with the sporadic detection by culture, underscoring the higher sensitivity of sequencing (Table 3).

**Table 3.** Comparison of genera detected by culture-based identification vs. 16S rRNA sequencing across individual TCM samples

Genus	*Areca catechu*	*Alpinia oxyphylla*	*E. caryophyllata*	*P. cablin*	*M. officinalis*
**Culture-based**					
*Enterobacter*	+	+	+	+	+
*Cronobacter*	+	−	−	−	−
*Leclercia*	+	−	−	−	−
*Klebsiella*	+	+	−	+	−
*Pantoea*	−	−	+	−	+
*Pseudomonas*	−	−	−	+	−
*Stenotrophomonas*	−	−	−	−	+
**16S rRNA seq**					
*Enterobacter*	6.8	10.8	0.3	2.3	0.0
*Cronobacter*	19.2	6.8	1.0	0.0	0.0
*Leclercia*	−	−	−	−	−
*Klebsiella*	38.3	6.7	0.0	1.7	0.0
*Pantoea*	83.5	164.5	97.2	342.5	21.8
*Pseudomonas*	3,553.0	2,665.2	1,480.7	1,921.7	1,935.2
*Stenotrophomonas*	18.2	25.5	13.2	16.3	3.8

+, Detected; –, undetected. The values represent the mean raw sequencing read counts (sequencing abundance) of ASVs assigned to each genus within a given type of TCM sample.

## Discussion

### Microbial contamination in herbal products is an important but often underrecognized public health concern

The complex biological matrices of herbs, coupled with risks introduced during cultivation, harvesting, processing and storage, create multiple opportunities for contamination. Recent outbreaks, such as the 2024 multistate *Salmonella* Typhimurium incident linked to organic basil in the USA, underscore the urgent need for vigilant monitoring and stringent quality control [25].

Many TCMs are derived from herbal plants, often carrying substantial microbial loads due to their inherent matrices and forms. For instance, rhizomes may harbour a high microbial load from the soil, while leaves, flowers and fruits can carry various micro-organisms from the air. Additionally, TCMs and their decoction pieces may be contaminated by microorganisms during planting, harvesting, processing and storage. Improper storage can further exacerbate microbial infections, such as *Escherichia coli*, *Salmonella*, *Shigella*, bile-resistant Gram-negative bacteria and *Candida albicans* [22, 26]. The microbial diversity and composition of *Areca catechu* and *Alpinia oxyphylla*, for example, appear similar, potentially due to shared growth environments or storage conditions (e.g. temperature, pH, nutrient availability and moisture) or a close historical and evolutionary relationship. In this study, bile-resistant Gram-negative bacteria, including *C. sakazakii*, *K. pneumoniae* and *Pseudomonas aeruginosa*, were identified in these TCM samples. *C. sakazakii*, a pathogenic bacterium of the *Enterobacteriaceae* family, can cause various intestinal diseases [27]. *K. pneumoniae*, also from the *Enterobacteriaceae* family, is a leading cause of community-acquired and hospital-acquired pneumonia, sepsis, meningitis and bacterial tissue abscesses [28]. *Pseudomonas aeruginosa*, widely distributed in nature and found on the skin, intestines and respiratory tract of healthy individuals, is a common opportunistic pathogen that can cause lung and urinary tract infections, as well as sepsis [29]. While high-temperature processing can eliminate most micro-organisms from these contaminated Chinese medicinal materials, some heat-resistant micro-organisms may persist [30]. These residual micro-organisms not only compromise the quality and efficacy of the medicinal materials but also pose a significant risk to drug safety, which poses a significant risk to drug safety.

### Culture-based identification

Culture-based identification is a traditional method for assessing the risk of microbial contamination in the microbial limit test of traditional Chinese medicine pieces [31]. The basic process involves ‘culture-enumeration and enrichment-culture-isolation and identification.’ This study selected five kinds of southern Chinese medicinal materials as research subjects. According to the 2020 edition of the Chinese Pharmacopoeia, these materials were tested for bile-resistant Gram-negative bacteria and *Salmonella*. The results showed that 40% of the samples contained bile-resistant Gram-negative bacteria, while *Salmonella* was not detected. Further identification revealed over ten types of contaminating micro-organisms from five families and seven genera in the five Chinese medicinal materials. The primary bile-resistant Gram-negative bacteria identified were from the *Enterobacteriaceae* and *Pseudomonas* families, including common pathogens such as *C. sakazakii*, *K. pneumoniae* and *Pseudomonas aeruginosa*. A study analysing the bile-resistant microbial communities in various Chinese herbal medicines using time-of-flight MS and high-throughput sequencing found that the main micro-organisms were from the *Enterobacteriaceae* and *Pseudomonadaceae* families, with *Enterobacteriaceae* having the highest detection rate at the genus level. The results of this study are consistent with those findings [32].

### Application of 16S rRNA high-throughput sequencing for microbial contamination detection in Chinese medicinal materials

To comprehensively analyse microbial contamination in Chinese medicinal materials, this study utilized the 16S rRNA high-throughput sequencing method in combination with traditional culture techniques. This approach allowed for the evaluation of microbial contamination risk and the analysis of microbial community diversity. Numerous studies have confirmed the feasibility of gene sequencing technology for detecting microbial contamination in Chinese medicinal materials and decoction pieces [22,33, 34]. In particular, the bacterial 16S rRNA high-throughput sequencing method is ideal for the rapid and comprehensive detection of both known and unknown bacterial species, as well as for assessing microbial diversity. Research has shown that *Proteobacteria* and *Actinobacteria* are the dominant microbial communities contaminating Chinese medicinal materials, with *Proteobacteria* accounting for the majority of microbial contamination [32].

While this molecular approach offers high sensitivity for taxonomic identification, it is important to acknowledge certain inherent limitations. First, 16S rRNA sequencing data only reflect the relative abundance profiles rather than absolute quantification. Therefore, while we identified the dominant bacterial DNA signatures, the actual biomass or total microbial load in the TCM samples remains unquantified. Future studies incorporating quantitative PCR or synthetic DNA spike-ins could provide a more precise assessment of the absolute microbial burden. Second, 16S sequencing is limited by its inability to distinguish cell viability, as it targets genomic DNA from both viable and non-viable (dead) cells. In contrast, fluorescent staining methods (e.g. Live/Dead cell staining) can directly differentiate viable from non-viable cells based on membrane integrity. Therefore, the detection of specific bacterial DNA signatures indicates the presence of genetic material but does not inherently confirm the viability or active pathogenicity of the identified taxa. Integrating fluorescent microscopy, flow cytometry or culture-based methods would complement sequencing data by providing real-time evidence of active microbial contamination.

### Comparison of 16S rRNA sequencing and culture-based identification

Through high-throughput sequencing, this study identified microbial community information from 26 major phyla and as many as 795 genera across 5 types of Chinese medicinal materials. In contrast, traditional microbial culture methods isolated only two phyla and seven genera of contaminating micro-organisms. Compared to traditional methods, the 16S rRNA high-throughput sequencing method provided a more comprehensive profile of contaminated microbial communities in Chinese medicinal materials and decoction pieces. The primary reason for this discrepancy is that the culture-based identification method relies heavily on predefined microbial species, specific culture conditions and the limited coverage of environmental strains in the VITEK-2 database. For instance, in this study, microbial colonies isolated on purple bile glucose agar and xylose lysine deoxycholate agar plates mainly belonged to the *Enterobacteriaceae* and *Pseudomonas* families. In contrast, the sequencing method does not have such limitations, demonstrating the superiority of high-throughput sequencing. Notably, DNA signatures of most micro-organisms identified by the culture-based identification method were also detected by the 16S rRNA sequencing method. However, the 16S rRNA sequencing method cannot fully replace the culture-based identification method. For example, *Enterobacter* in *M. officinalis* and *Leclercia* in *Areca catechu* were identified only by the culture-based identification method, but their DNA was not detected by 16S rRNA sequencing. This phenomenon may reflect methodological limitations of sequencing, including the 40-cycle PCR cap, primer bias, stringent data filtering thresholds, chimaera removal and insufficient sequencing depth, all of which can underestimate the low background levels of *Enterobacteriaceae* in southern medicinal materials. Although amplification may not reach the sequencing threshold, these bacteria can be readily detected when cultured under appropriate conditions using the VITEK-2 Compact system.

## Conclusion

For detecting microbial contamination in Chinese herbal medicines, both high-throughput sequencing technology and traditional methods have distinct advantages and limitations. The 16S rRNA high-throughput sequencing method can quickly and comprehensively analyse the diversity of contaminating micro-organisms in herbal medicine samples. However, it is constrained by factors such as the resolution capacity of the method itself and the level of microbial contamination in the samples. Traditional methods, on the other hand, can identify rare micro-organisms but are highly dependent on preset microbial species and specific culture conditions.

Therefore, this study directly compared the culture-based identification method recommended by the Chinese Pharmacopoeia (2020 edition) with the high-throughput 16S rRNA sequencing method. This comparison provided a more comprehensive evaluation of microbial contamination risks in Chinese herbal medicines, highlighted the differences in detection between the two methods and revealed a reduced detection rate for extremely low-abundance bacteria by culture-based approaches.

## Supplementary material

10.1099/jmm.0.002137 Supplementary Material 1.

## Abbreviations

ASVs: amplicon sequence variants
FDR: false discovery rate
NCBI: National Center for Biotechnology Information
NMDS: non-metric multidimensional scaling
PCoA: principal coordinate analysis
SRA: sequence read archive
TCMs: traditional Chinese medicines
TSB: tryptic soy broth
