# Supplementary material for: Microbial profiling of five traditional Chinese medicines: culture-based identification vs. 16S rRNA sequencing
Source: J Med Microbiol. 2026 Mar 31;75(3):002137. doi: 10.1099/jmm.0.002137 (PMC13038136; doi:10.1099/jmm.0.002137)
Supplement: Supplementary Material 1. [file jmm-75-02137-s001.pdf]

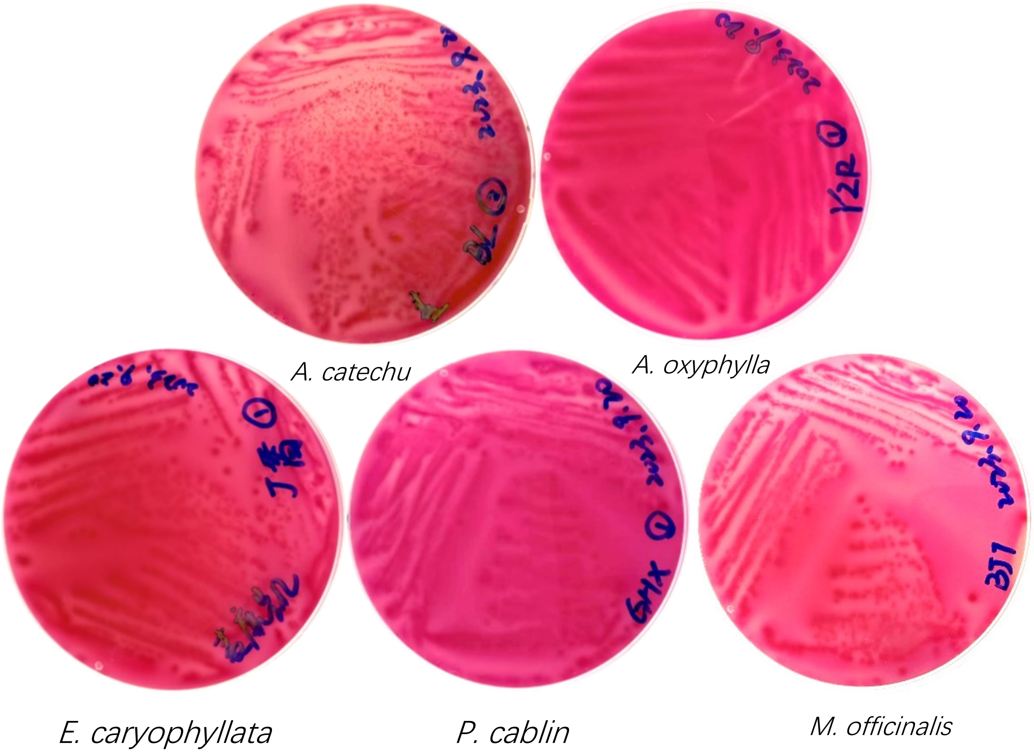

Figure S1. Representative images of microbial streaking from five TCMs on Violet Red Bile Glucose agar.

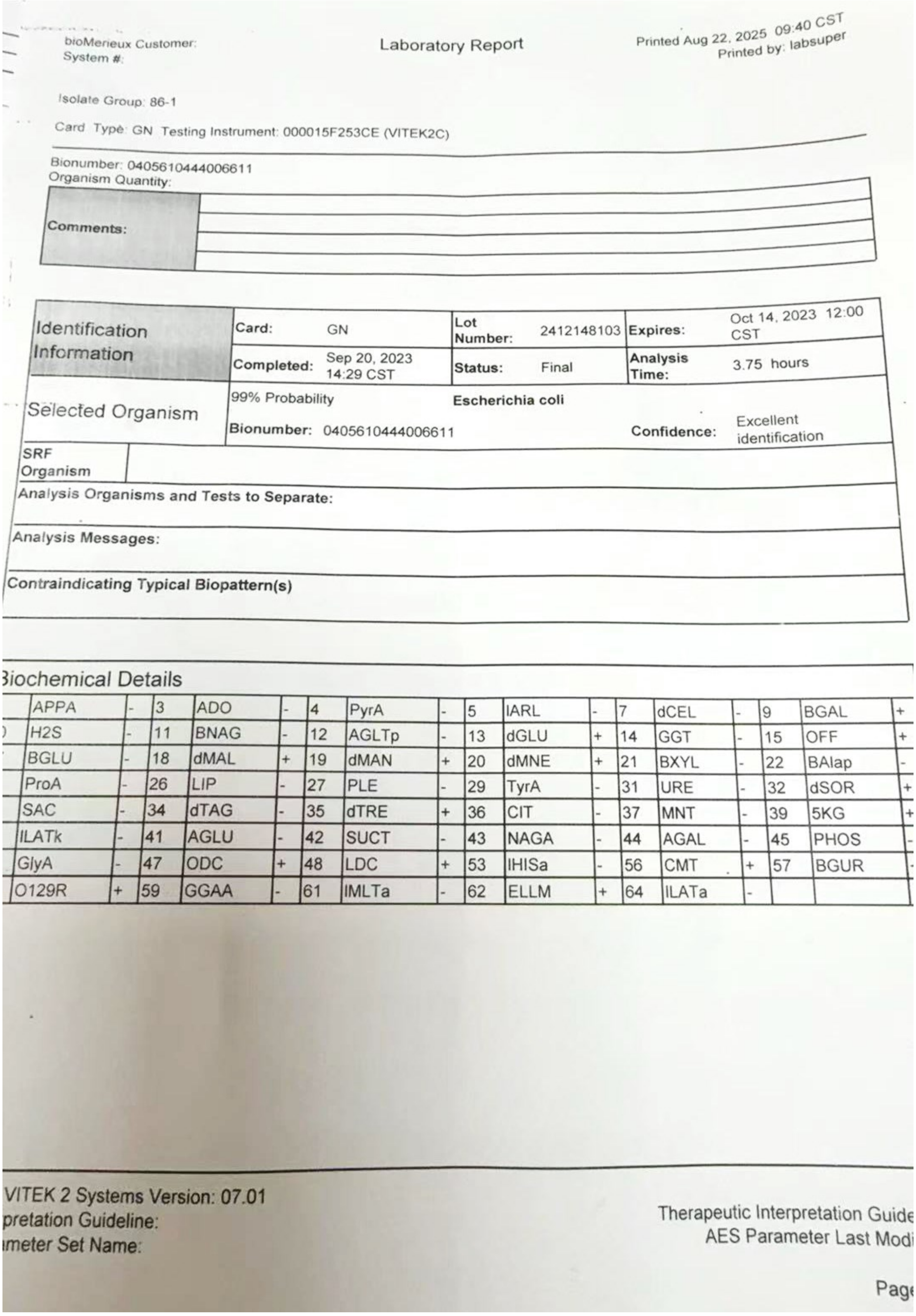

bioMerieux Customer:
System #:

Laboratory Report

Printed Aug 22, 2025 09:40 CST
Printed by: labsuper

Isolate Group: 86-1

Card Type: GN Testing Instrument: 000015F253CE (VITEK2C)

Bionumber: 0405610444006611
Organism Quantity:

Comments:	

Identification Information	Card:	GN	Lot Number:	2412148103	Expires:	Oct 14, 2023 12:00 CST
	Completed:	Sep 20, 2023 14:29 CST	Status:	Final	Analysis Time:	3.75 hours
Selected Organism	99% Probability		Escherichia coli			
	Bionumber:	0405610444006611			Confidence:	Excellent identification
SRF Organism						

Analysis Organisms and Tests to Separate:

Analysis Messages:

Contraindicating Typical Biopattern(s)

Biochemical Details

[illegible]	APPA	-	3	ADO	-	4	PyrA	-	5	IARL	-	7	dCEL	-	9	BGAL	+
[illegible]	H2S	-	11	BNAG	-	12	AGLTp	-	13	dGLU	+	14	GGT	-	15	OFF	+
[illegible]	BGLU	-	18	dMAL	+	19	dMAN	+	20	dMNE	+	21	BXYL	-	22	BAlap	-
[illegible]	ProA	-	26	LIP	-	27	PLE	-	29	TyrA	-	31	URE	-	32	dSOR	+
[illegible]	SAC	-	34	dTAG	-	35	dTRE	+	36	CIT	-	37	MNT	-	39	5KG	+
[illegible]	ILATk	-	41	AGLU	-	42	SUCT	-	43	NAGA	-	44	AGAL	-	45	PHOS	-
[illegible]	GlyA	-	47	ODC	+	48	LDC	+	53	IHISa	-	56	CMT	+	57	BGUR	-
[illegible]	O129R	+	59	GGAA	-	61	IMLTa	-	62	ELLM	+	64	ILATa	-			

VITEK 2 Systems Version: 07.01
pretation Guideline:
ımeter Set Name:

Therapeutic Interpretation Guide
AES Parameter Last Modi

Page

Figure S2. Representative laboratory report of microbial detection using the VITEK-2 Compact system.

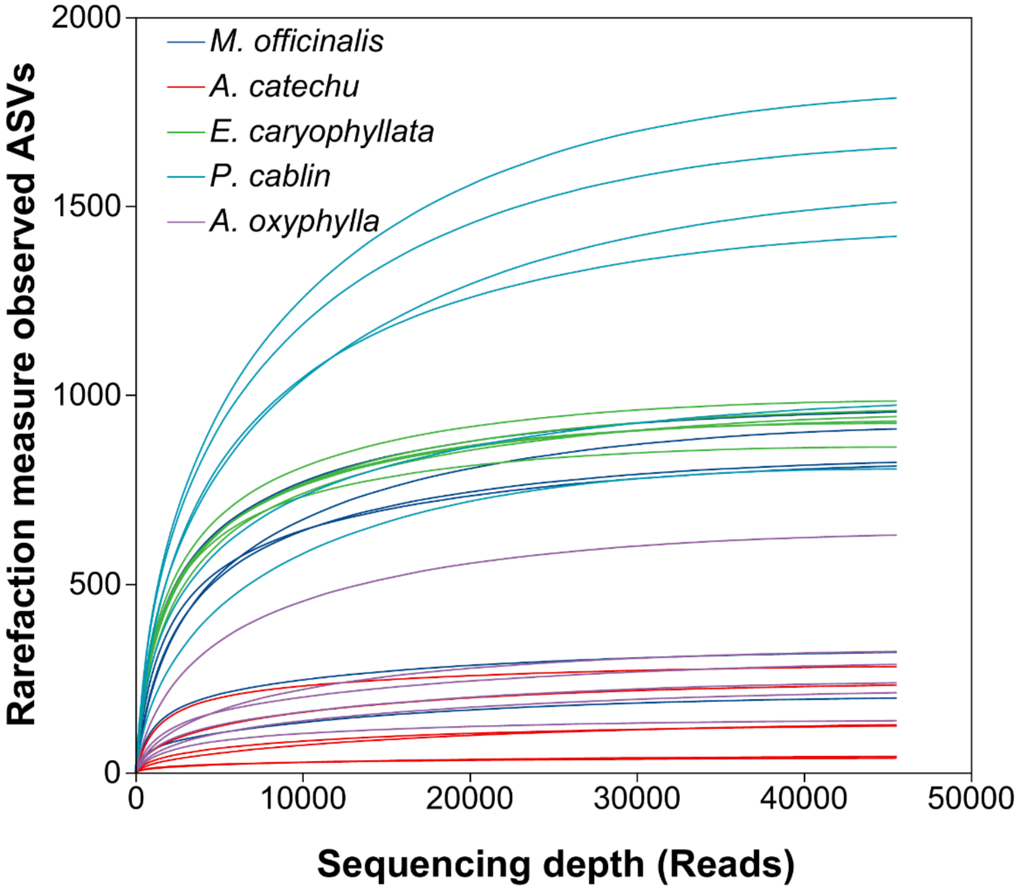

Figure S3. The rarefaction curves of ASVs in five types of Chinese Medicinal Materials. Amplicon sequence variants (ASVs) are inferred single DNA sequences obtained from high-throughput analysis of marker genes.

Table S1 The individual SRA accession numbers of 30 TCM sample

	Sample Name	BioSample accession	Experiment accession	Run accession	Notes
1	BJT1	SAMN42378088	SRX25244886	SRR29743918	*M. officinalis-1*
2	BJT2	SAMN42378089	SRX25244887	SRR29743917	*M. officinalis-2*
3	BJT3	SAMN42378090	SRX25244898	SRR29743906	*M. officinalis-3*
4	BJT4	SAMN42378091	SRX25244909	SRR29743895	*M. officinalis-4*
5	BJT5	SAMN42378092	SRX25244910	SRR29743894	*M. officinalis-5*
6	BJT6	SAMN42378093	SRX25244911	SRR29743893	*M. officinalis-6*
7	BL1	SAMN42378094	SRX25244912	SRR29743892	*A. catechu-1*
8	BL2	SAMN42378095	SRX25244913	SRR29743891	*A. catechu-2*
9	BL3	SAMN42378096	SRX25244914	SRR29743890	*A. catechu-3*
10	BL4	SAMN42378097	SRX25244915	SRR29743889	*A. catechu-4*
11	BL5	SAMN42378098	SRX25244888	SRR29743916	*A. catechu-5*
12	BL6	SAMN42378099	SRX25244889	SRR29743915	*A. catechu-6*
13	DX1	SAMN42378100	SRX25244890	SRR29743914	*E. caryophyllata-1*
14	DX2	SAMN42378101	SRX25244891	SRR29743913	*E. caryophyllata-2*
15	DX3	SAMN42378102	SRX25244892	SRR29743912	*E. caryophyllata-3*
16	DX4	SAMN42378103	SRX25244893	SRR29743911	*E. caryophyllata-4*
17	DX5	SAMN42378104	SRX25244894	SRR29743910	*E. caryophyllata-5*
18	DX6	SAMN42378105	SRX25244895	SRR29743909	*E. caryophyllata-6*
19	GHX1	SAMN42378106	SRX25244896	SRR29743908	*P. cablin -1*
20	GHX2	SAMN42378107	SRX25244897	SRR29743907	*P. cablin -2*
21	GHX3	SAMN42378108	SRX25244899	SRR29743905	*P. cablin -3*
22	GHX4	SAMN42378109	SRX25244900	SRR29743904	*P. cablin -4*
23	GHX5	SAMN42378110	SRX25244901	SRR29743903	*P. cablin -5*
24	GHX6	SAMN42378111	SRX25244902	SRR29743902	*P. cablin -6*
25	YZR1	SAMN42378112	SRX25244903	SRR29743901	*A. oxyphylla-1*
26	YZR2	SAMN42378113	SRX25244904	SRR29743900	*A. oxyphylla-2*
27	YZR3	SAMN42378114	SRX25244905	SRR29743899	*A. oxyphylla-3*
28	YZR4	SAMN42378115	SRX25244906	SRR29743898	*A. oxyphylla-4*
29	YZR5	SAMN42378116	SRX25244907	SRR29743897	*A. oxyphylla-5*
30	YZR6	SAMN42378117	SRX25244908	SRR29743896	*A. oxyphylla-6*
